# Cord blood stem cells revert glioma stem cell EMT by down regulating transcriptional activation of Sox2 and Twist1

**DOI:** 10.18632/oncotarget.367

**Published:** 2011-12-17

**Authors:** Kiran Kumar Velpula, Venkata Ramesh Dasari, Andrew J. Tsung, Dzung H. Dinh, Jasti S. Rao

**Affiliations:** ^1^ Department of Cancer Biology and Pharmacology, University of Illinois College of Medicine at Peoria, Peoria, Illinois, USA; ^2^ Department of Neurosurgery, University of Illinois College of Medicine at Peoria, Peoria, Illinois, USA

**Keywords:** Twist1, Sox2, human umbilical cord blood derived mesenchymal stem cells (hUCBSC), glioma stem cells (GSC), human Glioblastoma (hGBM), epithelial to mesenchymal transition (EMT), mesenchymal to epithelial transition (MET)

## Abstract

The dynamic nature of cancer stem cells that underlie metastasis or their ability to switch between different cellular identities, as in EMT and MET, has profound implications for cancer therapy. The functional relationship between molecules involved in cancer cell stemness and metastasis is not clear. In this regard, our studies on hGBM tissue grade IV specimens showed significant expression of Twist1 and Sox2, known mesenchymal and stemness related markers, respectively, indicating their association with glial tumor genesis and metastasis. The glioma stem cells obtained from CD133^+^ cells demonstrated increased expression of Twist1 and Sox2 accompanied by significant increase in the mesenchymal markers such as N-cadherin, vimentin and β-catenin. Our studies on glioma stem cells treatment with human umbilical cord blood derived- mesenchymal stem cells, showed down regulation of Twist1 and Sox2 proteins, apart from other mesenchymal stem cell markers. Based on the *in vitro* experiments and *in vivo* intracranial xenograft mouse model studies, we elucidated the potential therapeutic role of hUCBSC in suppressing glioma cancer stemness by the induction of MET.

## INTRODUCTION

Glioblastoma multiforme represents one of the most frequent brain tumors with high cellular complexity. Cancer stem cells (CSC) are cells within tumor cells that possess self-renewal capacity, causing heterogeneous lineages of cancer cells with distinct characteristics of tumor-initiating, invasion, neurosphere formation and differentiation [1-5] contributing tumor maintenance and recurrence [6, 7]. Tumor recurrence and metastasis are identified as the primary cause of treatment failure in several glioblastoma patients. It is thus essential to understand the molecular mechanisms that restore the GSCs balance towards differentiation.

Several investigators believed induction of EMT (epithelial to mesenchymal transition) is crucial for carcinogenesis and cancer progression [8, 9]. EMT of cancer cells leads to malignant carcinomas with an increased invasive or metastatic phenotype leading to tumor progression [10]. At the molecular level, EMT is interpreted by the down-regulation of epithelial markers and transcriptional induction of mesenchymal markers [11]. Transcription factors like Twist1 and SNAIL are known to be associated with cellular EMT, promoting cadherin switching. Twist1 is reported to be a master regulator of EMT and metastasis in breast, gastric, hepatocellular, prostate, and brain cancers including glioblastoma [11-21].

Further, growing evidences suggest that pathways regulating self-renewal are deregulated in cancer stem cells resulting in the continuous expansion of self-renewing cancer cells and tumor formation [22]. Sox2, commonly expressed by glioma cells and stem cells of the embryonic and adult brain [23] maintains proliferative potential of neural precursor cells, glioma-initiating cells and drives oncogenesis- associated aggressive tumor phenotype. Downregulation of Sox2 blocks proliferation and induces neuronal differentiation [24]. Variable percentage expression of Sox2 has been observed in gastric cancer, breast cancer, pancreatic cancer and glioblastoma [25], suggesting its relevance to cancer cell aberrant growth. Various experiments conducted on Sox2 have been shown to regulate the expression of other essential genes as well as its own transcription by positive feedback loops [26, 27]. Interestingly, several transcriptional networks with vital functions in neural stem cell populations appear to be co-expressed in gliomas. Studies are required to characterize the expression of different stem cell regulatory components in gliomas and to better understand their function in cancer progression.

In exploring new therapeutic strategies to counter glioblastoma progression, our previous studies have shown that human umbilical cord blood stem cells (hUCBSC) provides a promising platform to treat glioblastoma [28-30]. We investigated the co-regulatory role of Sox2 and Twist1 in GSC and their combinatorial effect on stemness maintenance and tumor progression. We also showed that restoration of glioma cancer cell EMT to MET pathway by hUCBSC treatment was accompanied by de-differentiation and dissemination of the tumor cells resulting in a less aggressive tumor phenotype. Our studies are focused on the role of hUCBSC in retarding GSC invasion and metastasis by suppressing the transcriptional activity and association of Sox2 and Twist1. To the best of our knowledge, this is the first study to discover that hUCBSC treatment per se might be significant enough to promote MET *in vivo*. However, further studies are needed to elucidate the underlying mechanisms.

## RESULTS

### Sox2 and Twist1 are expressed in human glioblastoma (hGBM) patient-derived specimens

**Figure 1:**
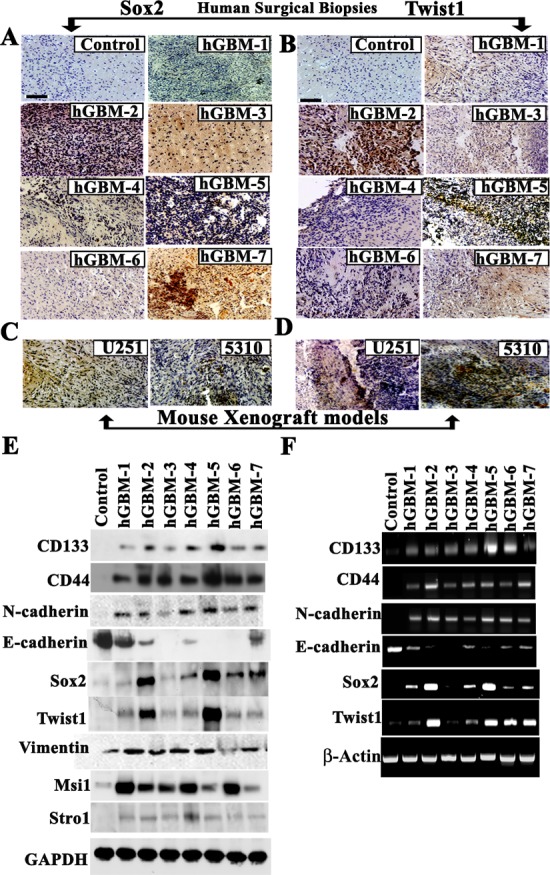
Primary glioblastoma tumors express mesenchymal makers Twist1 and Sox2 phenotype (A-B) Representative immunohistochemical staining of Twist1 and Sox2 in human GBM patient specimens and normal brain. Bar = 200 μm. Strong positivity of Twist1 and Sox2 was detected in five specimens (hGBMs 2,4,5,6 and 7). Negative staining was seen in normal human brain samples. (C-D) Specific expression of Twist1 and Sox2 in U251 and 5310 mouse xenografts models. Strong positivity of Twist1 and Sox2 was detected by H&E staining. Bar = 200 μm;. (E) GBM tissue specimen lysates for western blot analysis were prepared as described previously [30]. *In vivo* expression was studied by loading equal amounts of protein (40 μg) from tissue lysates onto 10-12% SDS-PAGE gels. (F) Semi-quantitative RT-PCR analysis was done to determine the expression of various stem cell markers in human GBM-derived biopsies. All determinations were done in duplicate.

Although earlier studies have indicated the independent expression of Sox2 [31-33] and Twist1 [12-14, 16, 18, 19] in different cancers including glioma [17, 25], the functional relationship of Sox2 and Twist1 together in GSC remains unknown. To evaluate this relationship, we examined seven tissue samples of grade IV gliomas (from surgical biopsy specimens) labeled with antibodies against Sox2 and Twist1. We detected high Sox2 and Twist1 expression levels in these samples with differential expression in non-tumor areas, suggesting their expression in cancerous tissues and not in normal brain tissue (Figs. 1A-1B). (Five hGBMs 2,4,5,6 and 7 of seven glioma specimens expressed variable levels of both Sox2 and Twist1). We next studied the global expression of these two regulatory molecules in different cancers. Twist1 expression was detected in medulloblastoma, meningioma, lung cancer, sarcoid lung cancer, and pancreatic cancer but not in prostate cancer (Supp. Fig. S1A) while Sox2 expression was seen in lung cancer, sarcoid lung cancer and meningioma but not in medulloblastoma, pancreatic and prostrate cancer (Supp. Fig. S1B). We also observed expression of Sox2 and Twist1 in U251 and 5310 parental cell lines (Figs. 1C-1D). Over expression of Sox2 and Twist1 in glioblastoma enhances tumor cell invasion and metastasis, thus supporting roles for Twist1 and Sox2 in glial tumorigenesis and progression [34, 35]. Our present data indicate that both Sox2 and Twist1 expression escorts glial tumorigenesis and that their higher expression levels may be associated with grade IV glial tumors.

To further characterize Sox2 and Twist1 association with malignant progression, we examined changes in protein and mRNA levels in hGBM samples. Western blotting along with RT-PCR analysis of these hGBM specimens demonstrated correlative expression of Sox2 and Twist1 (Figs. 1E-1F). Nonetheless, both Twist1 and Sox2 levels varied considerably among the seven hGBM samples studied but synergistic expression was observed between Twist1 and Sox2. The degree of malignancy of these GBM tumors may in part be reflected by the expression levels of both Twist1 and Sox2. Epithelial malignancy triggers the transition from an epithelial to a mesenchymal cell and is characterized by the loss of E-cadherin and gain of N-cadherin or vimentin expression. Immunoblot analysis showed increased expression of mesenchymal markers such as N-cadherin, Vimentin and decrease of E-cadherin. GSC markers, including CD133, CD44, Msi-1 and Stro-1, showed differential expression in the hGBM samples when compared to human control brain samples (Fig. 1E). These findings from western blot analysis were confirmed using RT-PCR to identify the expression of Sox2 and Twist1 along with other glioma stem cell markers in a panel of seven hGBM specimens (Fig. 1F).

### GSC characterization

**Figure 2:**
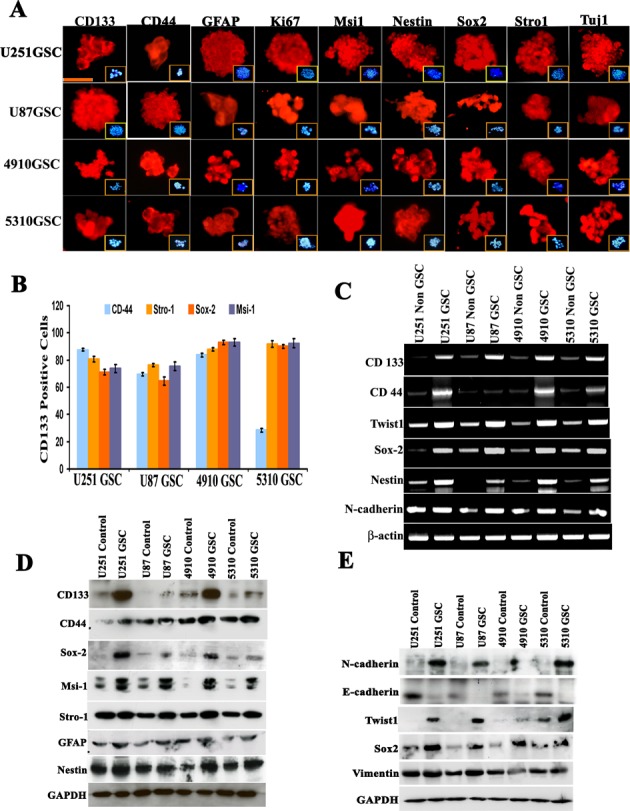
Evaluation of stem cell-like characteristics of U251, U87, 4910 and 5310 neurospheres (A) Immunocytochemical fluorescence analysis of U251, U87, 4910 and 5310 neurospheres (passage 10). The majority of neurospheres are immunopositive for CD133, CD44, GFAP, Ki-67, Msi-1, Nestin, Sox2, Stro-1 and Tuj-1. Inset pictures show DAPI (blue) staining. Bar = 100 μm. (B) Double positive co-expression of CD133^+^ GSCs isolated from U251, U87, 4910 and 5310 neurospheres were studied by flow cytometry. CD133-sorted cells from all four GSCs were labeled individually with CD44, Stro-1, Sox2 and Msi-1 with their respective antibodies. In all the experiments, CD133 was conjugated with Alexa Fluor-594 (red), and CD44, Stro-1, Sox2 and Msi-1 was conjugated with Alexa Fluor-488 (green). The experiment was performed in duplicate, and error bars represent standard deviation. (C) Expression levels of various glioma stem cell markers and other molecules were analyzed in U251, U87, 4910 and 5310 neurospheres in comparison to their respective non-GSC cells using semi-quantitative RT-PCR. β-actin served as an internal control for equal loading of the PCR products. Results presented are representative images of three independent experiments (n=3). (D-E) Western blot analysis of various neuronal and EMT makers in U251, U87, 4910 and 5310 neurospheres. Data presented here are a representation of three individual experiments (n=3). All cell lysates were obtained from cells at passage 10.

As shown by immunocytochemistry, GSCs obtained from U251, U87, 4910 and 5310 cells were positive for the GSC markers of proliferation, glial differentiation, and neuronal differentiation including CD133, CD44, GFAP, Ki-67, Msi-1, Nestin, Sox2, Stro-1 and Tuj1 (Fig. 2A), while the non-GSCs evaluated for the expression of the afore-mentioned markers showed reduced expression levels (Supp. Fig. S3D). To further assess the *in vitro* expression of Twist1 and Sox2 with regard to other GSC markers, we cultured CD133-sorted GSCs from U251, U87, 4910 and 5310 cell lines as neurospheres. Approximately 87.57% (U251 GSC), 69.58% (U87 GSC), 83.75% (4910 GSC) and 28.49% (5310 GSC) of CD133-labeled/sorted cells were positive for CD44; 80.5% (U251 GSC), 76.36% (U87 GSC), 87.8% (4910 GSC) and 91% (5310 GSC) of cells were positive for Stro1; 71.2% (U251 GSC), 64% (U87 GSC), 93.02% (4910 GSC) and 90% (5310 GSC) were positive for Sox2; and 73% (U251 GSC), 70.58% (U87 GSC), 73.75% (4910 GSC) and 82.49% (5310 GSC) were positive for Msi1 (Fig. 2B and Supp. Fig. S2). The GSCs obtained from U251, U87, 4910 and 5310 showed about 70-90% increase in the invasive potential while the non-GSCs showed 40-60% invasive potential by matrigel assay (Supp. Fig. S3A). In another experiment, hUCBSC co-culture challenged GSC- and their respective non GSC-induced microvessel formation (Supp. Fig. S3B). Alternatively, RT-PCR and western blot analysis confirmed abundant expression of aforementioned stem cell markers compared to their parent counterparts (Figs. 2C-2D). Earlier studies have shown that CD133 expression in tumor cells drives tumor progression [36], but the expression of other genes related to stemness on CD133-positive cells is not clearly understood. The increase in the expression of stemness related markers in GSCs (Figs. 2C-2D) was correlated with the increase in the expression of Sox2, Twist1, N-cadherin and Vimentin (Figs. 2C & 2E).

### Synergistic expression of Twist1 and Sox2 promotes EMT

**Figure 3:**
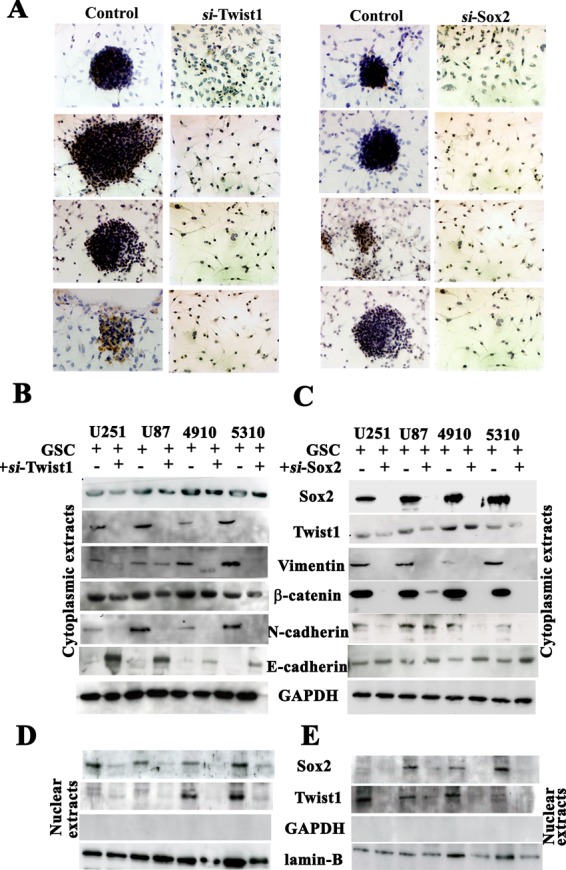
Analysis of U251, U87, 4910 and 5310 GSCs treated with Twist1- and Sox2- siRNA DAB immunocytochemistry was done to study the phenotypical changes of the U251, U87, 4910 and 5310 GSC when treated with (A) Twist1 siRNA and Sox2 siRNA. Cell lystates (40 μg protein for each sample) obtained by treating U251, U87, 4910 and 5310 GSCs with (B) siTwist and (C) siSox2 were loaded onto 8-12% SDS-PAGE gels, transferred onto nitrocellulose membranes, and probed with respective antibodies showing MET transition. (D-E) Nuclear extracts of the same samples were subjected to Western blotting (n=3). GSC (+) indicates the lysate obtained from scrambled vector in the immunoblots.

Previous reports indicate the role of Twist1 in increased tumor metastatic ability leading to the epithelial-to-mesenchymal transition [11]. In the present study, we found that down-regulation of either Twist1 or Sox2 in U251, U87, 4910 and 5310 GSC resulted in phenotypic change from neurospheres to differentiated colonies (Fig. 3A).

The increased expression of N-cadherin, vimentin, β-catenin and Sox2 in U251, U87, 4910 and 5310 control GSC when compared to the si-Twist-transfected samples is suggestive of the molecular alterations during mesenchymal transition (Figs. 3A & 3B). Apart from the EMT markers in Twist1 knockdown cells, we also observed a significant decrease in Sox2 levels, which is the first finding of this sort. To study in detail, our hypothesis that Twist1 regulates Sox2 expression, we transfected U251, U87, 4910 and 5310 GSCs with si-Sox2. We observed increased expression levels of E-cadherin but reduced N-cadherin, vimentin, β-catenin and Twist1 expression in the si-Sox2 transfectants (Fig. 3C). Nuclear extracts of U251, U87, 4910 and 5310 GSCs from both si-Twist1 and si-Sox2 transfected samples demonstrated reduced expression levels when compared to their respective control GSCs (Figs. 3D-3E). Collectively, the above data suggests that the expression of both Twist1 and Sox2 in accord may be necessary to maintain the stemness and induce EMT in GSC.

### Expression studies of Twist1 and Sox2 in si-Twist, si-Sox2 transfected GSC

**Figure 4:**
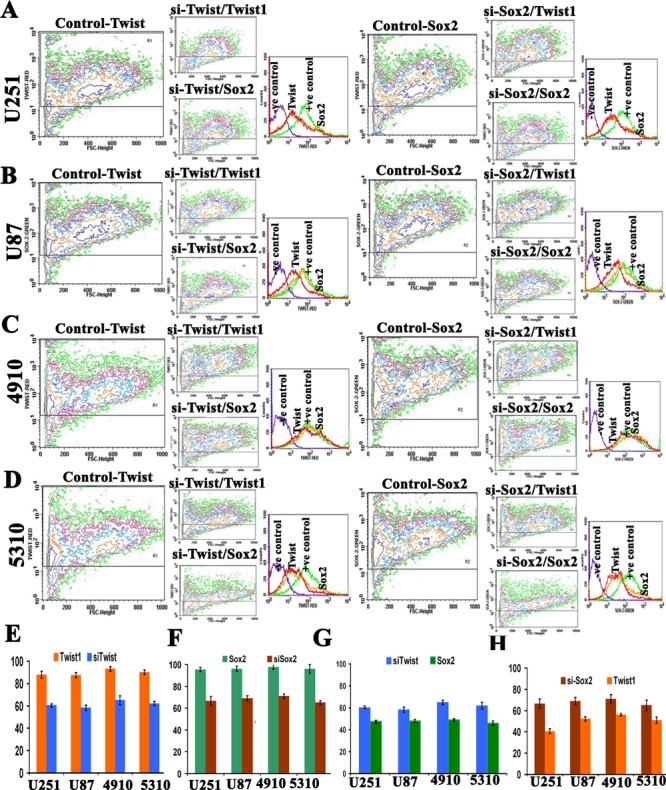
Effect of siTwist and siSox2 on Twist1 and Sox2 expression using frequency distribution contour plots Approximately 1×10^5^ U251 (A), U87 (B), 4910 (C), and 5310 (D) cells were transfected with either siTwist or si-Sox2. Samples were harvested after 72 hrs. siTwist1 and siSox2 expression was determined by staining with the mAb to human Twist1, Sox2 or isotype control mouse or goat IgG_1_, and then followed by a secondary antibody goat anti-mouse IgG or donkey anti-goat IgG conjugated to Alexa Fluor red or green dye. The background was subtracted using different isotypic controls for respective antibodies. In overlays under Figures 4A-4D, purple color represents the negative control, green color indicates the siTwist or siSox2 treatment, orange color represents Sox2 expression, and red color indicates Twist1 expression. Bar graph (E) represents the expression level of Twist1 in siTwist1 knock down (F) represents the expression level of Sox2 in siSox2 knockdown (G) represents the expression level of Sox2 in siTwist1 knock down while (H) represents the percent expression levels of Twist1 in siSox2 knock down as quantified from the above contour plots. Cells were analyzed with a FACSCalibur flow cytometer and CellQuest software.

To further confirm expression levels of Twist1 and Sox2 in both si-Twist1 and si-Sox2 transfected GSCs, we did FACS analysis. Contour plots were plotted to study frequency distribution of the expression levels of Twist1 and Sox2. si-Twist1 reduced the expression levels of both Twist1 and Sox2, while si-Sox2 reduced both Sox2 and Twist1 expression levels in all four different GSCs studied (Figs. 4A-4D). Overlay of the dot plots, shows reduced expression levels of Sox2 and Twist1 as observed in contour plots to provide clear understanding of the synergistic phenomenon. The percent expression of Twist1 after cells were treated with si-Twist was observed to be reduced to 43%, 40%, 28% and 35% in U251, U87, 4910 and 5310 GSCs, respectively. The percent expression of Sox2 after cells were treated with si-Twist was observed to be reduced to 25%, 16%, 18% and 14% in U251, U87, 4910 and 5310 GSCs, respectively (Figs. 4E-4F). Similarly, the percent expression of Sox2 after cells were treated with si-Sox2 was reduced to 29%, 27.8%, 29% and 31% in U251, U87, 4910 and 5310 GSCs, respectively. The percent expression of Twist after cells were treated with si-Sox2 was observed to be reduced to 18.6%, 19%, 16% and 16% in U251, U87, 4910 and 5310 GSCs, respectively (Figs. 4G-4H). These results are in agreement with our earlier findings that knockdown of Twist1 led to reduced expression of Sox2 and similarly, knockdown of Sox2 decreased Twist1 expression. These results suggest that their combinatorial effect may contribute to the maintenance of stemness and metastatic ability of the GSC.

### Treatment of GSC with hUCBSC induces MET

**Figure 5:**
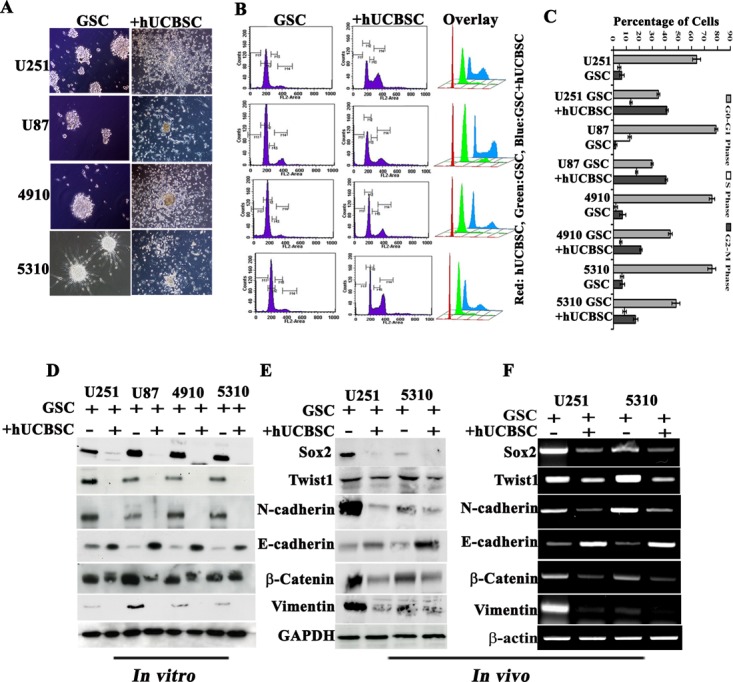
Effect of hUCBSC on GSC (A) Co-culture experiments showing induction of MET transition by hUCBSC in GSCs. (B) Various stages of cell cycle of GSCs influenced by hUCBSC. In all of the GSCs, cell cycle was arrested at the G2-M stage. (C) Bar graph showing the results of (B). Western blot analysis showing MET pathway molecules in U251, U87, 4910 and 5310 GSCs when co-cultured with hUCBSC (D) *in vitro* (72 hrs) and (E) *in vivo*. (F) Semi-quantitative RT-PCR analysis showing MET transition of GSCs.

Previously, we have shown that hUCBSC regulates glioma tumor progression [28, 29]. Taking cue from our previous findings, we tried to evaluate the efficacy of hUCBSC against U251, U87, 4910 and 5310 GSCs. To start with, 1×10^6^ GSCs were co-cultured with 1×10^6^ hUCBSC (1:1 ratio) for 72 hrs. We observed that hUCBSC co-culture retrogress the aggressive mesenchymal glioma stem cells to their epithelial mode via differentiation reverting back to the parent phenotype (Fig. 5A). To further analyze the kinetics of hUCBSC-induced differentiation and proliferation, different phases of the cell cycle were determined by FACS analysis after 72 hrs. U251, U87, 4910 and 5310 control GSCs showed 64%, 79.01%, 75.75%, and 75.69% of the G_0_-G_1_ phase; 4.3%, 12% 1.89%, and 6.1% of the S phase; and 6.38%, 1.69%, 7.09%, and 6.82% of the G_2_-M phase, respectively. In contrast, hUCBSC-treated cells showed 34.6%, 29.75%, 43.81%, and 43.82% of the G_0_-G_1_ phase; 13.3%, 18% 6.89%, and 8.1% of the S phase; and 41.22%, 40.59%, 21.17%, and 17% of the G_2_-M phase, respectively (Figs. 5B-5C). To determine if the treatment of hUCBSC resulted in a decrease in the invasive ability of GSCs, we monitored the effect of serum-induced invasion through Matrigel. GSC/ non-GSCs migrated aggressively through Matrigel pores while hUCBSC treatment reduced the invasive potential by around 40-70% (Supp. Fig. S3A). Similarly, angiogenic tube formation was reduced both in GSC/non-GSCs when co-cultured with hUCBSC (Supp. Fig. S3B).

Since it is understood that Twist1 and Sox2 regulate the metastatic ability of tumor cells and their involvement in stemness preservation, we analyzed the hUCBSC co-culture treated GSC for Sox2 and Twist1 expression. Immunoblotting analysis demonstrate that treatment of U251, U87, 4910 and 5310 GSC with hUCBSC reduced N-cadherin, β-catenin, vimentin, Sox2 and Twist1 expression, with increased E-cadherin levels indicative of MET course (Fig. 5D). To further explore the effect of hUCBSC on GSCs, we looked for changes in cellular functions frequently associated with EMT by hUCBSC treatment. We examined the expression levels of the above mentioned proteins in U251 and 5310 GSC xenografts by western blot analysis; the expression levels of mesenchymal markers were decreased upon hUCBSC treatment (Fig. 5E). RT-PCR results confirmed our above results obtained from western blot analysis (Fig. 5F).

### hUCBSC treatment inhibits Sox2 and Twist1 expression in the intracranial GSC induced tumors of mice

**Figure 6:**
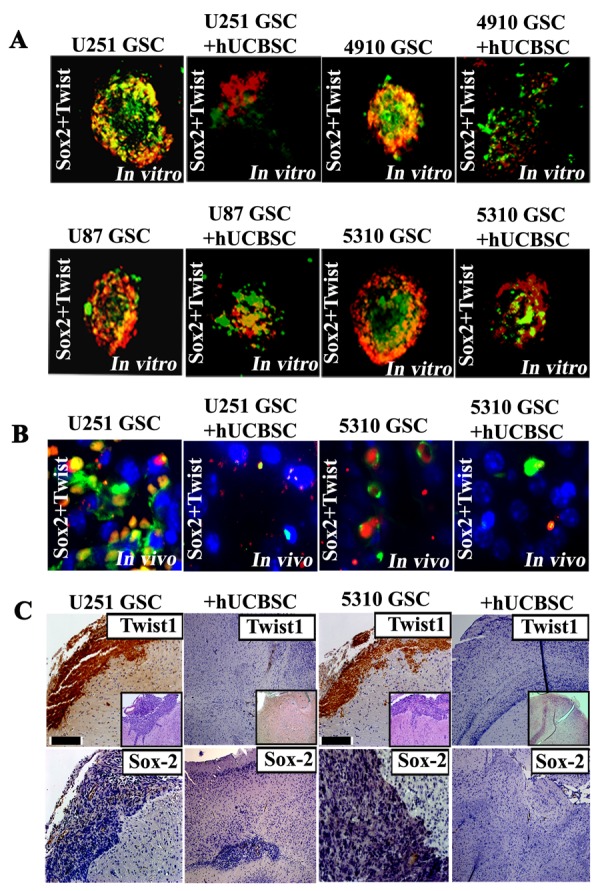
Twist1 co-expresses with Sox2 in U251, U87, 4910 and 5310 GSCs (A) Immunostaining analysis from neurospheres generated from GSC-enriched populations (U251, U87, 4910 and 5310 cells) shows Twist1 staining that appears to correlate with high Sox2 expression. Photographs depicted show Twist1 (red) is co-expressed/associated with Sox2 (green). All nuclei are counterstained with DAPI in blue. Scale bars represent 100 μm. (B) Immunostaining of GBM mouse xenografts (U251 and 5310) demonstrate that Twist1 (red) is expressed in the necrotic area and is co-expressed with Sox2 (green). Scale bars represent 50 μm. DAB immunohistochemical analysis of Twist1 and Sox2 expression in U251 GSC and 5310 GSC xenografts in nude mice brains. (C) Representative microphotographs of U251 and 5310 GSCs injected intracranially, which show infiltrative/migratory nature of xenograft-derived tumors. Insets in figures are representative H&E stained sections. Immunohistochemical staining was done using specific antibodies for Twist1 and Sox2 (dark brown in color). Scale bar represents 100 μm.

To determine the analeptic of hUCBSC on GSC tumor formation *in vivo*, athymic nude mice were injected with U251 and 5310 GSCs (1×10^5^) in the left frontal lobe. Mice injected with U251 and 5310 GSCs showed detectable tumor characteristics, such as hunch back appearance, paralyzed limbs, severe weight loss and neurologic pain within 12-18 days. After three weeks, an equal number of hUCBSC (1×10^5^) were injected into the right lobe of the mice, already having GSC induced tumors. The hUCBSC treatment showed regression in GSC induced tumors and tumor development. Mice treated with only hUCBSC did not form tumors for 120 days (Data not shown). Pathological examination of GSC induced tumors in the nude mice showed a vast necrotic area associated with the tumor tissues; hUCBSC treatment significantly reduced GSC induced tumor volumes (Supp. Fig. S3C). The tumor volumes were reduced to 65-75% in U251 and 75-90% in 5310 GSC- treated mice upon hUCBSC treatment as evidenced by H&E staining (Fig. 6C). To corroborate our findings and to extend our results from hGBM tissue specimens, we analyzed the expression of both Twist1 and Sox2 by immunohistochemistry. Adjacent sections were used to study the expression of both Twist1 and Sox2. High expression of Twist1 and Sox2 was observed in the necrotic regions of U251 and 5310 GSC induced tumor, while hUCBSC-treated U251 and 5310 xenografts showed reduced or no expression of either Twist1 or Sox2 in individual experiments (Fig 6C).

As evidenced by the significant increase in the expression of both Twist1 and Sox2 in the necrotic tumor areas, and their co-regulatory effect in siRNA transfectants, we next asked whether this expression is the co-regulatory mechanism necessitated by the interaction between Twist1 and Sox2. Initial experiments in this direction were targeted at neurospheres at the *in vitro* level. Control GSCs obtained from U251, U87, 4910 and 5310 showed clear interaction and co-localization between Twist1 and Sox2 while hUCBSC treatment showed decreased expression and co-localization of both these proteins (Fig. 6A). These results prompted us to study co-localization of these proteins in an *in vivo* xenograft model. U251 and 5310 GSC control tissue samples showed co-localization of Sox2 with Twist1 which was significantly reduced upon hUCBSC treatment (Fig. 6B). The association of transcription factors, Sox2 (responsible for stemness) and Twist1 (responsible for EMT) thus provides a platform to correlate the stem cell pathway with EMT pathway, which can be further studied to address potential targets for glioblastoma cure.

## DISCUSSION

**Table 1:** PCR primers

Gene	Forward Primer	Reverse Primer
**CD133**	5'CCAAGTTCTACCTCATGTTTGG 3'	5'ACCAACAGGGAGATTGCAAAGC3'
**CD44**	5' AATCCCTGCTACCAATATGGACT 3'	5' AGCCTTCAGAATGATTTGGGTC 3'
**N-cadherin**	5'CAACTTGCCAGAAAACTCCAGG 3'	5' ATGAAACCGGGCTATCTGCTC 3'
**E-cadherin**	5' CGAGAGCTACACGTTCACGG 3'	5' GGCCTTTTGACTGTAATCACACC 3'
**Sox2**	5' CACAACTCGGAGATCAGCAA 3'	5' CTCCGGGAAGCGTGTACTTA 3'
**Twist1**	5'CGGACAAGCTGAGCAAGATT 3'	5' CCTTCTCTGGAAACAATGAC 3'
**Nestin**	5' GGCATCCTCACCCTGAAGTA 3'	5' CTTGGGGTCCTGAAAGCTG 3'
**b-catenin**	5'GAAACGGCTTTCAGTTGAG 3'	5' CTGGCCATATCCACCAGAGT 3'
**Vimentin**	5'GAACGCCAGATGCGTGAAATG3'	5'CCAGAGGGAGTGAATCCAGATTA3'
**b-actin**	5'GGCATCCTCACCCTGAAGTA3'	5'GGGGTGTTGAAGGTCTCAAA3'

Cancer stem cells render abnormal growth, frequency and radio-resistance in malignant glioblastoma. Therapeutic strategies in treating glioblastoma require a better understanding of the mechanisms that promote stem cell maintenance that sustain tumor growth. Elias, et al. [34] proposed neural precursor cells as the likely targets for malignant transformation and showed that deregulation of the self-renewal capacity of normal stem cells resulted in stable augmentation of self-renewing cancer cells and tumor formation. Investigations to study this tumorigenic process will provide a powerful tool to uncover new targets for glioma therapeutic intervention. On par with the available evidence, it is understood that cancer stem cells (CSC) play an important role in tumor initiation, growth, invasion and recurrence. Therefore, identification and study of CSC will provide insights into better understanding of brain tumors and their cellular targets. In this study, we have obtained GSCs from U251, U87, 4910 and 5310 cell lines using the surface protein CD133 and FACS sorting as reported previously [37-39].

In the present study, we show that malignant human grade IV gliomas display significant expression of Twist1 and Sox2. Twist1 expression was observed in hGBMs as well as in medulloblastoma, meningioma, lung and pancreatic cancer specimens, while Sox2 expression was observed in hGBM specimens along with that of meningioma, lung and pancreatic cancers. These results indicate the participation of both Twist1 and Sox2 in the genesis of a wide range of neuroectodermal tumors. Twist1 encourages cancer cell survival and tumor progression by increasing resistance to cytotoxic therapies resulting in increased cellular proliferation [40]. Twist1 is considered to be a key player of EMT, a metastatic cascade in which epithelial cells take over a mesenchymal phenotype [10]. EMT phenotype expresses cancer stem-like cells signatures associated with tumor recurrence and a drug-resistant phenotype [41]. Immunoblotting analysis conducted on the hGBM specimens have shown reduced epithelial E-cadherin expression and upregulated mesenchymal N-cadherin, vimentin, β-catenin and Twist1, suggesting the EMT mode. High expression levels of other stem cell markers such as CD44, Msi-1, Stro-1, and EMT markers, such as Sox2, Twist1, vimentin suggested an invasive phenotype. The synergistic up-regulation of Twist1 and Sox2 expression in hGBM specimens may instigate E-cadherin replacement by N-cadherin expression triggering cadherin switching [42, 43].

Numerous lines of evidences suggest that the Twist1 regulates programmed cell death and induces apoptosis [44, 45]. Moreover, it has been observed that transfection of Bel7402 (highly expressing Twist1) cells with Twist1 shRNA plasmid resulted in suppression of Twist1 expression and subsequent inhibition of cell invasion and migration *in vitro*, indicating its possible role in tumor cell plasticity [46]. The widespread expression of Twist1 in various cancer phenotypes suggests its action upon a diverse set of downstream target genes, depending upon the tissue, to elicit a variety of cellular responses. The chimeric nature of beta-Helix loop Helix (bHLH) proteins, such as Twist1 are implicated to possibly combine heterodimerically with other bHLH proteins to activate or repress diverse downstream targets generating different types of biological responses [47]. Cimadamore *et al.*, [48] reported that knockdown of Sox2, the SRY (sex-determining region)-box 2 gene, resulted in downregulation of proneural bHLH transcription factors.

Twist1-mediated molecular changes in our study provided important insight into its role in understanding mesenchymal change with genes related to EMT being upregulated in U251, U87, 4910 and 5310 GSCs. Since we observed the co-expression of Sox2 along with Twist1, further studies are warranted to examine their role in acquiring an invasive malignant phenotype. Knockdown of Twist1 reduced the expression levels of Sox2, while knockdown of Sox2 reduced the expression levels of Twist1. In both experiments, increased expression of E-cadherin was observed, which suggests that both Twist1 and Sox2 function in harmony and that their concurrence is essential for the maintenance of tumor plasticity. Inhibition of Twist1 or Sox2 expression by their specific siRNAs resulted in differentiation of GSC formation and growth and is suggestive of the above discussed mechanism.

Targeted migration of mesenchymal stem cells (MSCs) to tumors makes them a very promising strategy for anti-tumor therapy [49]. Previously, we have demonstrated the potential therapeutic applications of hUCBSC in the treatment of gliomas [28, 29]. In addition, with increased understanding of the mechanistic underpinnings of MSC action, the potential for enhancing MSC-targeted therapies appears promising. This report provides a strong evidence that hUCBSC inhibit the metastatic and invasive potential of U251, U87, 4910 and 5310 GSCs. Co-culture of hUCBSC with these GSCs changed their morphology and clearly reduced their migration and invasion abilities. We also showed that hUCBSC induced MET in U251, U87, 4910 and 5310 GSCs, which might be responsible for inhibition of metastasis-related abilities.

It appears that hUCBSC counteract and repress Twist1 expression by increasing E-cadherin levels, a phenomenon generally observed at sites of EMT. Our present data demonstrated the ability of hUCBSC to inhibit GSC phenotypes in co-culture experiments both *in vitro* and *in vivo*. Immunoblotting experiments showed a decrease in the expression levels of both Twist1 and Sox2 along with other EMT markers, suggesting that hUCBSC treatment reversed the EMT to MET. Based on all our findings, we propose that hUCBSC inhibit GSC invasion, migration by suppressing key molecules involved in EMT. These actions resulted in induction of a MET state, which is a key step in reversal and treatment of the cellular metastatic process.

In conclusion, for the first time, we showed that interactive and co-regulatory role of Twist1 and Sox2 necessary to maintain glioma stemness was inhibited by hUCBSC. Here, hUCBSC was administered to study key aspects in treating the glioma stem cell machinery. We engineered hUCBSC near the tumor site and observed that hUCBSC are effective in regressing tumor growth in the intracranial xenograft mouse model. Based on our findings, we conclude that hUCBSC can be used as a means of therapy and will be of great interest for the clinical application of stem cell-based cancer therapy.

## MATERIALS AND METHODS

### Tumor samples

Glioblastoma specimens were obtained during autopsies of glioblastoma patients within 24 hrs of death or from patients who underwent surgery at Saint Francis Medical Center (Peoria, IL). All samples were collected under protocols approved by the UICOMP (Peoria, IL) Institutional Review Board. All tumors without prior evidence of progression from a lower-grade tumor were clinically classified as primary glioblastoma.

### Culture of hUCBSC

Human umbilical cord blood stem cells were isolated as described previously (See supplemental methods) [29].

### Enrichment for glioma stem cells

Human glioma cell lines parental and non-GSC (U251 and U87) and xenografts (4910 and 5310) were cultured using conventional tissue culture media: DMEM/RPMI-1640 (Hyclone, Logan, UT) supplemented with 10% fetal bovine serum (Gibco BRL, Grand Island, NY), 100 units/mL penicillin/streptomycin (Lonza, Walkersville, MD). GBM neurospheres of U87, U251, and xenografts 4910, 5310 were cultured in neurobasal medium supplemented with N2 (1%), B27 (1%), 20 ng/mL of bFGF and EGF, 10 ng/mL of LIF (Millipore, Billerica, MA)[50]. Experiments for the present study were conducted using the neurospheres obtained between the 5-14^th^ passages.

### Immuno- cyto- histochemical staining

Experiments were performed to detect the expressi1 on of various stem cell markers CD133, CD44, Msi-1, Stro1, Nestin, Sox2, Ki-67, GFAP and Tuj-1 in both GSC and non-GSCs (see Supplemental methods).

### Semi-Quantitative RT-PCR

Total RNA (5 μg) from cells, brains or tumor samples extracted using Qiagen RNeasy mini kit (Valencia, CA) was reverse-transcribed using the Transcriptor First Strand cDNA Synthesis Kit (Roche, Indianapolis, IN). (See Supplementary methods)

### CD133 sorting, cell cycle analysis

GSCs obtained from U251, U87, 4910 and 5310 cells dissociated by trituration into single cell suspension were incubated for 60 min at 4°C with anti-CD133 phycoerythrin (PE)-conjugated antibody (clone AC133-PE, mouse IgG1; Miltenyi Biotec, Bergisch Gladbach, Germany) according to the manufacturer's instructions. PE-conjugated mouse IgG1 isotype antibody was used as control. The cells re-suspended in 0.5 mL PBS-BSA were analyzed and sorted by flow cytometry. Acquisition was performed on a FACSCalibur flow cytometer (Becton Dickinson, San Jose, CA) and viable cells were analyzed with CellQuest software. In a separate experiment, the CD133-positive cells were labeled with anti-CD44, anti-Stro-1, anti-Msi-1 and anti-Sox2 in independent experiments using a similar staining protocol. The control for each sample was prepared identically except that an isotype-specific antibody was used. Progression through different cell cycle phases in U251, U87, 4910 and 5310 cells alone and in co-culture with hUCBSC for 72 hrs was monitored by flow cytometric analysis of the DNA content of cell populations stained with propidium iodide (BioSure, Grass Valley, CA).

### Matrigel invasion and *In vitro* angiogenesis assay

The Matrigel invasion assay and tumor conditioned medium-induced microtubule network formation were studied in U251, U87, 4910 and 5310 GSC, non-GSC alone or in co-culture with hUCBSC (See supplemental methods and materials).

### siRNA-based knockdown of Twist1 and Sox2

The siRNA targeting human *Twist1* (sc-38604), *Sox2* (sc-38408) and scrambled negative control RNA (Santa Cruz, CA). were used to transfect U251, U87, 4910 and 5310 GSCs (2.5×10^5^ cells) using 6 μL of Fugene HD reagent (Roche, Indianapolis, IN). The siRNA/Fugene HD complex was added to the GSCs and the transfection was carried out for 48-72 hrs. Nuclear extracts were prepared from these siRNA-transfected samples using a nuclear extraction kit from Panomics, Inc. (Fremont, CA) according to the manufacturer's instructions.

### Contour plot FACS analysis

Twist1 and Sox2 expression was measured by intracellular protein staining using antibodies specific for human Twist1 and Sox2 (Santa Cruz Technologies, Santa Cruz, CA). The Twist1 siRNA- or Sox2 siRNA-transfected cells were incubated for 1 hr with gene-specific Twist1 or Sox2. Twist1 expression in si-Sox2-treated samples and Sox2 expression in si-Twist1 treated samples were studied by measuring cellular fluorescence using the FACSCalibur flow cytometer (Becton Dickinson, San Jose, CA).

### Western blotting

GBM parental or stem cells alone or in co-culture with hUCBSC for 72 hrs were collected, lysed in RIPA buffer and then resolved via SDS-PAGE (See supplemental methods).

### Intracranial administration of GSC and hUCBSC

Glioma stem cells were injected intracerebrally into the left side of the brain of nude mice with 10 μL aliquots of U251 and 5310 GSC (1×10^5^) under isofluorane anesthesia with the aid of a stereotactic frame. Three weeks after tumor implantation, hUCBSC were injected towards the right side of the brain in 1:1 ratio. Six mice from each group were sacrificed by cardiac perfusion with 4% formaldehyde in PBS, and paraffin sections were prepared. Sections were stained with H&E to visualize tumor cells and to examine tumor volume. The sections were blindly reviewed and scored semi-quantitatively for tumor size. The average visible tumor area per section integrated to the number of sections was calculated using the formula 1/6 II (Rmax) × (Rmin)2, where Rmax and Rmin are the maximum and minimum tumor radii, respectively. The calculated tumor volume was compared between controls and treated groups.

## Supplementary Methods and Figures





## References

[1] Galli R, Binda E, Orfanelli U, Cipelletti B, Gritti A, De Vitis S, Fiocco R, Foroni C, Dimeco F, Vescovi A (2004). Isolation and characterization of tumorigenic, stem-like neural precursors from human glioblastoma. Cancer Research.

[2] Hemmati HD, Nakano I, Lazareff JA, Masterman-Smith M, Geschwind DH, Bronner-Fraser M, Kornblum HI (2003). Cancerous stem cells can arise from pediatric brain tumors. Proceeding of the National Academy of Science USA.

[3] Li Z, Bao S, Wu Q, Wang H, Eyler C, Sathornsumetee S, Shi Q, Cao Y, Lathia J, McLendon RE, Hjelmeland AB, Rich JN (2009). Hypoxia-inducible factors regulate tumorigenic capacity of glioma stem cells. Cancer Cell.

[4] Singh SK, Hawkins C, Clarke ID, Squire JA, Bayani J, Hide T, Henkelman RM, Cusimano MD, Dirks PB (2004). Identification of human brain tumour initiating cells. Nature.

[5] Vescovi AL, Galli R, Reynolds BA (2006). Brain tumour stem cells. Nature Reviews Cancer.

[6] Bao S, Wu Q, McLendon RE, Hao Y, Shi Q, Hjelmeland AB, Dewhirst MW, Bigner DD, Rich JN (2006). Glioma stem cells promote radioresistance by preferential activation of the DNA damage response. Nature.

[7] Hambardzumyan D, Squatrito M, Holland EC (2006). Radiation resistance and stem-like cells in brain tumors. Cancer Cell.

[8] Wu Y, Zhou BP (2008). New insights of epithelial-mesenchymal transition in cancer metastasis. Acta Biochimica et Biophysica. Sinica. (Shanghai).

[9] Yang J, Weinberg RA (2008). Epithelial-mesenchymal transition: at the crossroads of development and tumor metastasis. Developmental. Cell.

[10] Thiery JP (2002). Epithelial-mesenchymal transitions in tumour progression. Nature Reviews Cancer.

[11] Yang J, Mani SA, Donaher JL, Ramaswamy S, Itzykson RA, Come C, Savagner P, Gitelman I, Richardson A, Weinberg RA (2004). Twist, a master regulator of morphogenesis, plays an essential role in tumor metastasis. Cell.

[12] Casas E, Kim J, Bendesky A, Ohno-Machado L, Wolfe CJ, Yang J (2011). Snail2 is an essential mediator of Twist1-induced epithelial mesenchymal transition and metastasis. Cancer Research.

[13] Jeronimo C, Esteller M (2010). DNA methylation markers for prostate cancer with a stem cell twist. Cancer Prevention Research. (Phila).

[14] Kwok WK, Ling MT, Lee TW, Lau TC, Zhou C, Zhang X, Chua CW, Chan KW, Chan FL, Glackin C, Wong YC, Wang X (2005). Up-regulation of TWIST in prostate cancer and its implication as a therapeutic target. Cancer Research.

[15] Lee TK, Poon RT, Yuen AP, Ling MT, Kwok WK, Wang XH, Wong YC, Guan XY, Man K, Chau KL, Fan ST (2006). Twist overexpression correlates with hepatocellular carcinoma metastasis through induction of epithelial-mesenchymal transition. Clinical Cancer Research.

[16] Ma JL, Han SX, Zhu Q, Zhao J, Zhang D, Wang L, Lv Y (2011). Role of Twist in vasculogenic mimicry formation in hypoxic hepatocellular carcinoma cells in vitro. Biochemical and Biophysical Research Communication.

[17] Mikheeva SA, Mikheev AM, Petit A, Beyer R, Oxford RG, Khorasani L, Maxwell JP, Glackin CA, Wakimoto H, Gonzalez-Herrero I, Sanchez-Garcia I, Silber JR, Horner PJ, Rostomily RC (2010). TWIST1 promotes invasion through mesenchymal change in human glioblastoma. Molecular Cancer.

[18] Mironchik Y, Winnard PT, Vesuna F, Kato Y, Wildes F, Pathak AP, Kominsky S, Artemov D, Bhujwalla Z, Van DP, Burger H, Glackin C, Raman V (2005). Twist overexpression induces in vivo angiogenesis and correlates with chromosomal instability in breast cancer. Cancer Research.

[19] Ru GQ, Wang HJ, Xu WJ, Zhao ZS (2011). Upregulation of twist in gastric carcinoma associated with tumor invasion and poor prognosis. Patholology Oncology Research.

[20] Yuen HF, Chua CW, Chan YP, Wong YC, Wang X, Chan KW (2007). Significance of TWIST and E-cadherin expression in the metastatic progression of prostatic cancer. Histopathology.

[21] Zhang Z, Xie D, Li X, Wong YC, Xin D, Guan XY, Chua CW, Leung SC, Na Y, Wang X (2007). Significance of TWIST expression and its association with E-cadherin in bladder cancer. Human Pathology.

[22] Ge Y, Zhou F, Chen H, Cui C, Liu D, Li Q, Yang Z, Wu G, Sun S, Gu J, Wei Y, Jiang J (2010). Sox2 is translationally activated by eukaryotic initiation factor 4E in human glioma-initiating cells. Biochemical and Biophysical Research Communication.

[23] Gangemi RM, Griffero F, Marubbi D, Perera M, Capra MC, Malatesta P, Ravetti GL, Zona GL, Daga A, Corte G (2009). SOX2 silencing in glioblastoma tumor-initiating cells causes stop of proliferation and loss of tumorigenicity. Stem Cells.

[24] Fang X, Yoon JG, Li L, Yu W, Shao J, Hua D, Zheng S, Hood L, Goodlett DR, Foltz G, Lin B (2011). The SOX2 response program in glioblastoma multiforme: an integrated ChIP-seq, expression microarray, and microRNA analysis. BMC. Genomics.

[25] Schmitz M, Temme A, Senner V, Ebner R, Schwind S, Stevanovic S, Wehner R, Schackert G, Schackert HK, Fussel M, Bachmann M, Rieber EP, Weigle B (2007). Identification of SOX2 as a novel glioma-associated antigen and potential target for T cell-based immunotherapy. Britich Journal of Cancer.

[26] Boer B, Kopp J, Mallanna S, Desler M, Chakravarthy H, Wilder PJ, Bernadt C, Rizzino A (2007). Elevating the levels of Sox2 in embryonal carcinoma cells and embryonic stem cells inhibits the expression of Sox2:Oct-3/4 target genes. Nucleic Acids Research.

[27] Boyer LA, Lee TI, Cole MF, Johnstone SE, Levine SS, Zucker JP, Guenther MG, Kumar RM, Murray HL, Jenner RG, Gifford DK, Melton DA, Jaenisch R, Young RA (2005). Core transcriptional regulatory circuitry in human embryonic stem cells. Cell.

[28] Dasari VR, Velpula KK, Kaur K, Fassett D, Klopfenstein JD, Dinh DH, Gujrati M, Rao JS (2010). Cord Blood Stem Cell-Mediated Induction of Apoptosis in Glioma Downregulates X-Linked Inhibitor of Apoptosis Protein (XIAP). PLoSOne.

[29] Velpula KK, Dasari VR, Tsung AJ, Gondi CS, Klopfenstein JD, Mohanam S, Rao JS (2011a). Regulation of glioblastoma progression by cord blood stem cells is mediated by downregulation of cyclin D1. PlosOne.

[30] Velpula KK, Dasari VR, Tsung AJ, Dinh DH, Rao JS (2011b). Transcriptional repression of MAD-MAX complex by human umbilical cord blood stem cells downregulates ERK in glioblastoma. Stem Cells and Development.

[31] Chen Y, Shi L, Zhang L, Li R, Liang J, Yu W, Sun L, Yang X, Wang Y, Zhang Y, Shang Y (2008). The molecular mechanism governing the oncogenic potential of SOX2 in breast cancer. Journal of Biological Chemistry.

[32] Park ET, Gum JR, Kakar S, Kwon SW, Deng G, Kim YS (2008). Aberrant expression of SOX2 upregulates MUC5AC gastric foveolar mucin in mucinous cancers of the colorectum and related lesions. International Journal of. Cancer.

[33] Sanada Y, Yoshida K, Ohara M, Oeda M, Konishi K, Tsutani Y (2006). Histopathologic evaluation of stepwise progression of pancreatic carcinoma with immunohistochemical analysis of gastric epithelial transcription factor SOX2: comparison of expression patterns between invasive components and cancerous or nonneoplastic intraductal components. Pancreas.

[34] Elias MC, Tozer KR, Silber JR, Mikheeva S, Deng M, Morrison RS, Manning TC, Silbergeld DL, Glackin CA, Reh TA, Rostomily RC (2005). TWIST is expressed in human gliomas and promotes invasion. Neoplasia.

[35] Wegner M, Stolt CC (2005). From stem cells to neurons and glia: a Soxist's view of neural development. Trends in Neuroscience.

[36] Lathia JD, Gallagher J, Heddleston JM, Wang J, Eyler CE, MacSwords J, Wu Q, Vasanji A, McLendon RE, Hjelmeland AB, Rich JN (2010). Integrin alpha 6 regulates glioblastoma stem cells. Cell Stem Cell.

[37] Ieta K, Tanaka F, Haraguchi N, Kita Y, Sakashita H, Mimori K, Matsumoto T, Inoue H, Kuwano H, Mori M (2008). Biological and genetic characteristics of tumor-initiating cells in colon cancer. Annals of Surgical Oncology.

[38] Liu G, Yuan X, Zeng Z, Tunici P, Ng H, Abdulkadir IR, Lu L, Irvin D, Black KL, Yu JS (2006). Analysis of gene expression and chemoresistance of CD133+ cancer stem cells in glioblastoma. Molecular Cancer.

[39] Pfenninger CV, Roschupkina T, Hertwig F, Kottwitz D, Englund E, Bengzon J, Jacobsen SE, Nuber UA (2007). CD133 is not present on neurogenic astrocytes in the adult subventricular zone, but on embryonic neural stem cells, ependymal cells, and glioblastoma cells. Cancer Research.

[40] Wang X, Ling MT, Guan XY, Tsao SW, Cheung HW, Lee DT, Wong YC (2004). Identification of a novel function of TWIST, a bHLH protein, in the development of acquired taxol resistance in human cancer cells. Oncogene.

[41] Mani SA, Guo W, Liao MJ, Eaton EN, Ayyanan A, Zhou AY, Brooks M, Reinhard F, Zhang CC, Shipitsin M, Campbell LL, Polyak K, Brisken C, Yang J, Weinberg RA (2008). The epithelial-mesenchymal transition generates cells with properties of stem cells. Cell.

[42] Alexander NR, Tran NL, Rekapally H, Summers CE, Glackin C, Heimark RL (2006). N-cadherin gene expression in prostate carcinoma is modulated by integrin-dependent nuclear translocation of Twist1. Cancer Research.

[43] Maeda M, Johnson KR, Wheelock MJ (2005). Cadherin switching: essential for behavioral but not morphological changes during an epithelium-to-mesenchyme transition. Journal of Cell Science.

[44] Valsesia-Wittmann S, Magdeleine M, Dupasquier S, Garin E, Jallas AC, Combaret V, Krause A, Leissner P, Puisieux A (2004). Oncogenic cooperation between H-Twist and N-Myc overrides failsafe programs in cancer cells. Cancer Cell.

[45] Yousfi M, Lasmoles F, El Ghouzzi V, Marie PJ (2002). Twist haploinsufficiency in Saethre-Chotzen syndrome induces calvarial osteoblast apoptosis due to increased TNFalpha expression and caspase-2 activation. Human. Molecular Genetics.

[46] Sun T, Zhao N, Zhao XL, Gu Q, Zhang SW, Che N, Wang XH, Du J, Liu YX, Sun BC (2010). Expression and functional significance of Twist1 in hepatocellular carcinoma: its role in vasculogenic mimicry. Hepatology.

[47] Franco HL, Casasnovas J, Rodriguez-Medina JR, Cadilla CL (2011). Redundant or separate entities?--roles of Twist1 and Twist2 as molecular switches during gene transcription. Nucleic Acids Research.

[48] Cimadamore F, Fishwick K, Giusto E, Gnedeva K, Cattarossi G, Miller A, Pluchino S, Brill LM, Bronner-Fraser M, Terskikh AV (2011). Human ESC-derived neural crest model reveals a key role for SOX2 in sensory neurogenesis. Cell Stem Cell.

[49] Kosztowski T, Zaidi HA, Quinones-Hinojosa A (2009). Applications of neural and mesenchymal stem cells in the treatment of gliomas. Expert Review of Anticancer Therapy.

[50] Lee J, Kotliarova S, Kotliarov Y, Li A, Su Q, Donin NM, Pastorino S, Purow BW, Christopher N, Zhang W, Park JK, Fine HA (2006). Tumor stem cells derived from glioblastomas cultured in bFGF and EGF more closely mirror the phenotype and genotype of primary tumors than do serum-cultured cell lines. Cancer Cell.

