# Supplementary material for: Cord blood stem cells revert glioma stem cell EMT by down regulating transcriptional activation of Sox2 and Twist1
Source: Oncotarget. 2011 Dec 17;2(12):1028–42. doi: 10.18632/oncotarget.367 (PMC3282065; doi:10.18632/oncotarget.367)
Supplement: Supplementary file 1 [file oncotarget-02-1028-s001.pdf]

Supplemental methods:

Culture of hUCBSC. Human umbilical cord blood stem cells were isolated using Ficoll-Paque (GE Health Care, Piscataway, NJ) density gradient centrifugation. The isolated cells were plated in 100-mm plates in DMEM knockout medium (Invitrogen, Carlsbad, CA) supplemented with 10% fetal bovine serum, 10% knockout serum (Hyclone, Logan, UT), and 1% penicillin/streptomycin. When the adherent cells reached 20% to 30% confluence, they were supplemented with Mesencult medium (Stem Cell Technologies, Vancouver, Canada) containing mesenchymal stem cell stimulatory supplements (human) (Stem Cell Technologies, Vancouver, Canada) and 1% penicillin/streptomycin (Invitrogen, Carlsbad, CA). For co-culture experiments, hUCBSC and glioma stem cells were cultured for 72 hrs at a ratio of 1:1.

Matrigel invasion assay. The Matrigel invasion assay, which is used to study cell invasive potential, was performed in cell culture medium supplemented with 10% FBS added to the lower chamber to act as a chemoattractant. U251, U87, 4910 and 5310 glioma stem cells, alone or in co-culture with hUCBSC cells, were seeded at a density of 2×10^5 cells/well onto the upper inserts and incubated at 37°C. After 24 hrs, the non-invasive cells were removed from the upper surface of the separating membrane by gentle scrubbing with a cotton swab, and the invading cells were fixed in 100% methanol and stained with Hema-3.

***In vitro* angiogenesis assay.** Tumor conditioned medium-induced microtubule network formation was determined by adding conditioned medium from U251, U87, 4910 and 5310 glioma stem cells and hUCBSC-treated glioma cells to HMEC (human microvascular endothelial cells) derived from dermis and provided by Dr. Francisco J. Candal (Centers for Disease Control and Prevention, Atlanta, GA). After 48 hrs in the treated media, dead floating cells were washed away and the cells were stained with Hema-3 to show angiogenesis.

Western blotting. GBM parental or stem cells alone or in co-culture with hUCBSC for 72 hrs were collected and lysed in RIPA buffer [50 mmol/mL Tris-HCl (pH 8.0), 150 mmol/mL NaCl, 1% IGPAL, 0.5% sodium deoxycholate, 0.1% SDS] containing 1 mM sodium orthovanadate, 0.5 mM PMSF, 10 μg/mL aprotinin, and 10 μg/mL leupeptin and then resolved via SDS-PAGE. After transfer onto nitrocellulose membranes, blots were blocked with 5% non-fat dry milk in PBS and 0.1% Tween-20. Blots were incubated with respective primary antibodies, followed by incubation with a HRP-conjugated secondary antibody. Immunoreactive bands were visualized using chemiluminescence ECL western blotting detection reagents on Hyperfilm-MP autoradiography film (Amersham, Piscataway, NJ). GAPDH or β-actin antibody was used to verify equal loading of proteins in all lanes. A similar protocol was repeated for all of the different treatments.

Immuno- cyto, -histochemical staining. Immunocytochemistry was performed to detect the expression of various stem cell markers CD133, CD44, Msi-1, Stro1, Nestin, Sox2, Ki-67, GFAP and Tuj-1 in both GSC and non-GSCs. U251, U87, 4910 and 5310 GSC plated on slides coated with poly-lysine (Sigma, St. Louis, MO) were fixed for 30 min with 4% formaldehyde solution and permeablized with 0.3% Triton X-100/PBS for 5 min followed by incubation with 10% goat serum for 30 min. Paraffin embedded human glioblastoma tissues along with other cancer specimens were de-paraffinized for antigen

retrieval in 0.01 mol/L sodium citrate buffer (pH 6.0). After washing with PBS, cells were incubated for 2 hrs or overnight with respective primary antibodies. Cells/tissues were either incubated with Alexa Fluor-conjugated secondary antibodies (1:200; Molecular Probes, Eugene, OR) / respective secondary antibodies for 1 hr. The cells were counterstained with DAPI to identify the nuclei using confocal microscopy. Staining in tissue sections was visualized by DAB-based horseradish peroxidase (HRP) reaction and counterstained with hematoxylin and eosin (H&E).

Semi-Quantitative RT-PCR. Total RNA (5 μg) from cells, brains or tumor samples extracted using Qiagen RNeasy mini kit (Valencia, CA) was reverse-transcribed using the Transcriptor First Strand cDNA Synthesis Kit (Roche, Indianapolis, IN). Go Taq PCR master mix (Promega, Madison, WI) was used for template amplification. Primer sequences were designed from human GenBank sequences using Primer 3 software (v.0.4.0). PCR primers are listed in table 1.Reverse transcriptase PCR was set up using the PCR cycle [95˚C for 5 min, (95˚C for 45 sec, 55-60˚C for 45 sec, and 72˚C for 45 sec) x 35 cycles, 72˚C for 10 min].
