# Supplementary material for: Cord blood stem cells revert glioma stem cell EMT by down regulating transcriptional activation of Sox2 and Twist1
Source: Oncotarget. 2011 Dec 17;2(12):1028–42. doi: 10.18632/oncotarget.367 (PMC3282065; doi:10.18632/oncotarget.367)
Supplement: Supplementary file 2 [file oncotarget-02-1028-s002.pdf]

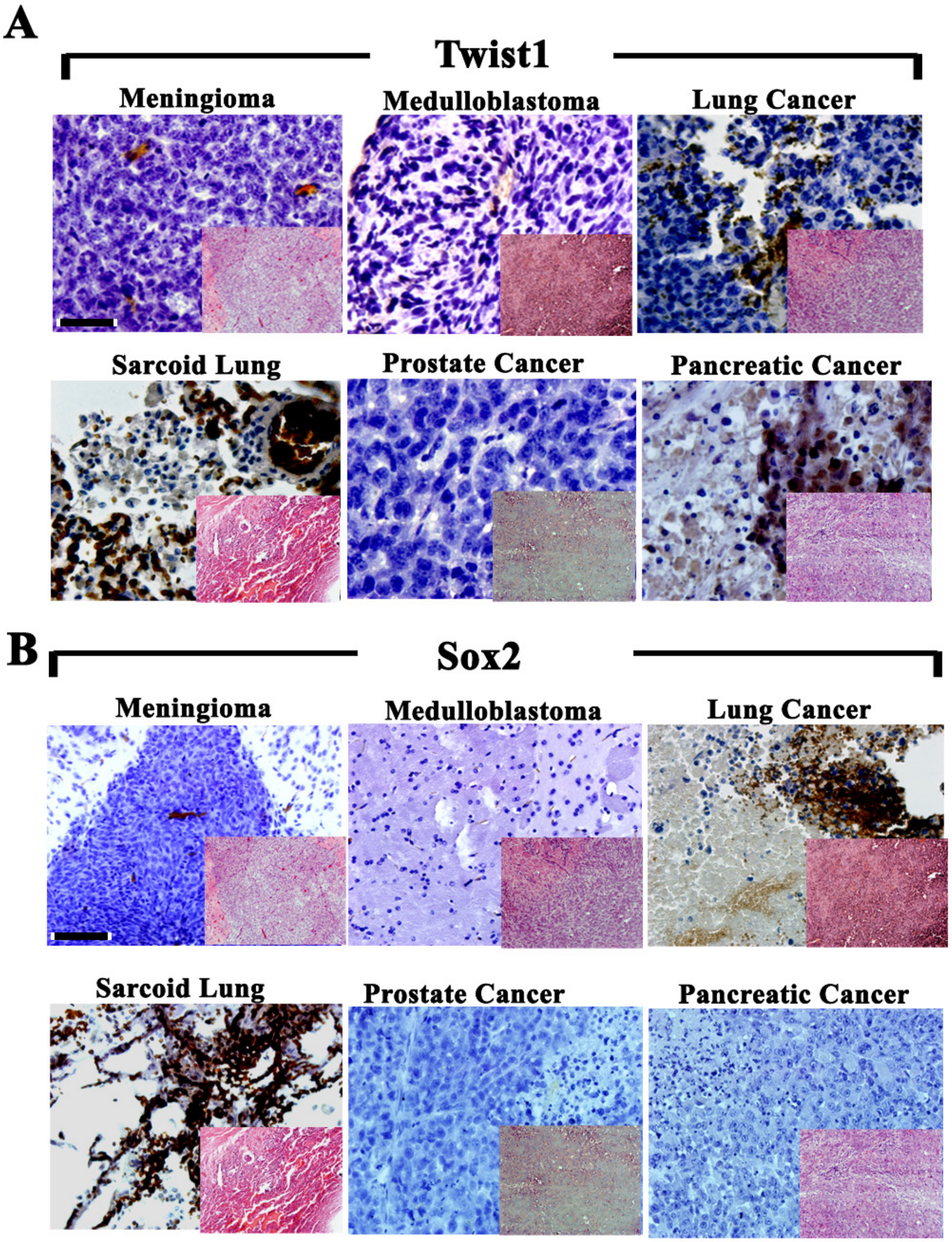

Supplementary Figure S1: Twist1 and Sox2 expression patterns in various cancers as shown by immunostaining. H&E histology is represented in the insets. Twist1 (A) and Sox2 (B) immunostaining demonstrates high staining in lung, sarcoid lung, and pancreatic cancers while occasional faint staining is observed in meningioma and medulloblastoma. Negative staining is seen in prostate cancer. Abundant pronounced nuclear staining is present in all of the tumors evaluated. Scale = 100 µm.

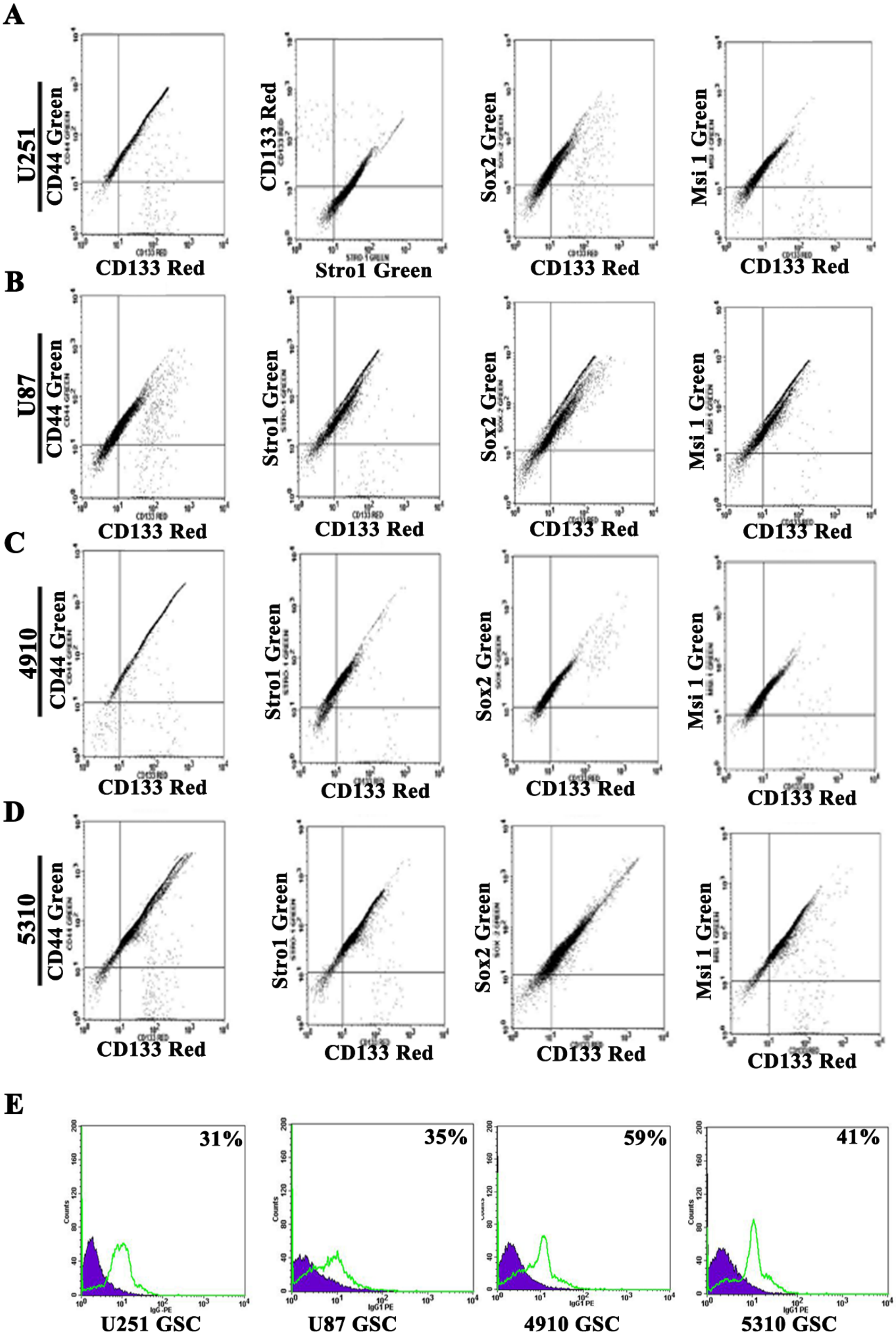

Supplementary Figure S2: Expression of MSC-associated surface markers in glioblastoma cells. Co-expression of CD44, Stro-1, Msi-1, and Sox2 in CD133-positive GSC neurospheres were analyzed using dual fluorescence-activated cell sorting analysis in (A) U251, (B) U87, (C) 4910, and (D) 5310 GSCs (E) Flow cytometry analysis for expression of CD133. Expression of CD133 in U251, U87, 4910 and 5310 GSCs was determined by staining with the PE-labeled anti-CD133 or isotype control mouse IgG_1. The background has been subtracted using isotopic control.

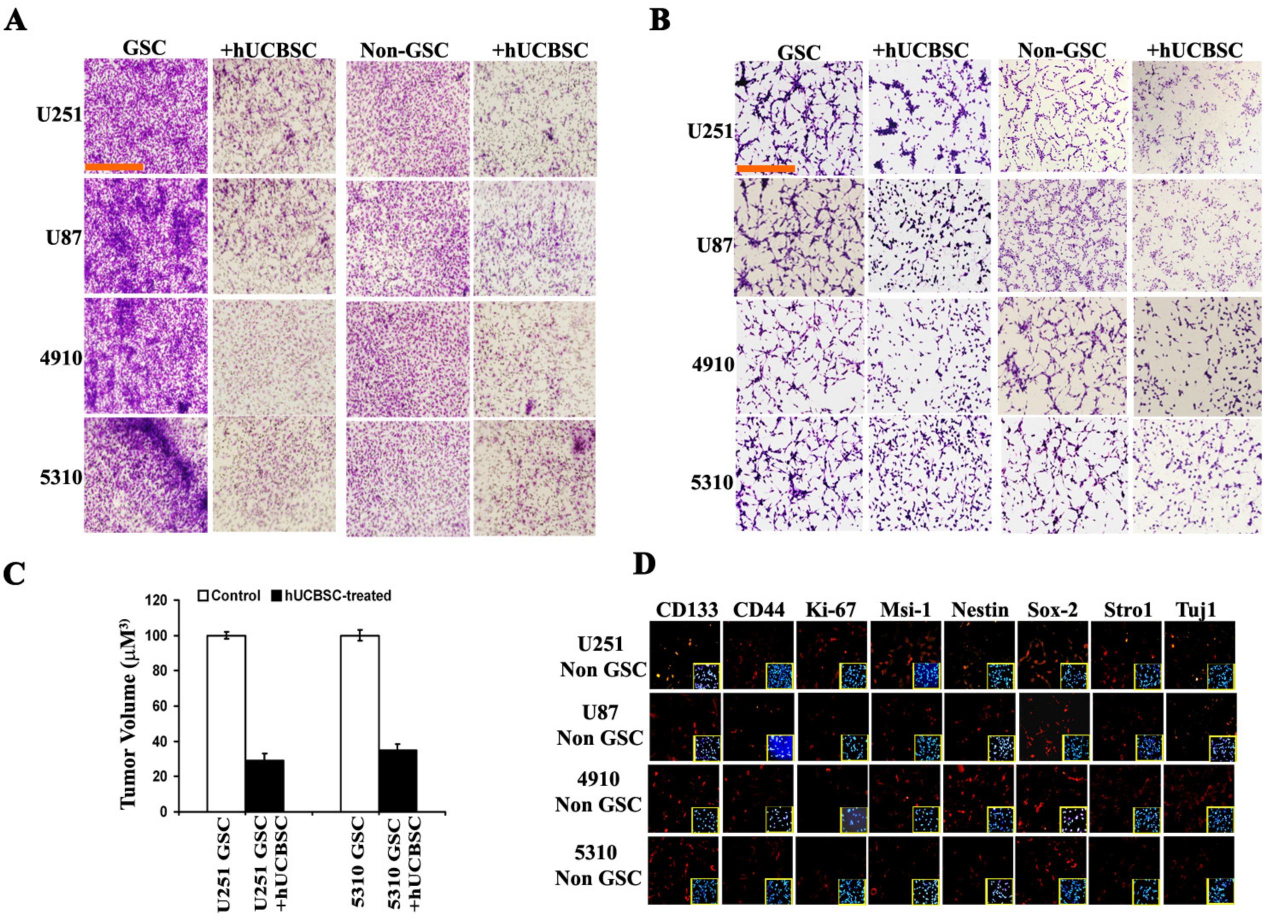

Supplementary Figure S3: Matrigel invasion assay. (A) U251, U87, 4910 and 5310 GSCs, their respective non-GSCs co-cultured with hUCBSC for 72 h were allowed to pass through Matrigel membrane. After 24 hrs, the cells were stained with Hema-3 and the images were taken using bright-field microscopy. (B) Conditioned medium collected from GSCs, non-GSCs and their co-cultures with hUCBSC were subjected to angiogenic vessel formation in HMEC cells for 48 hrs. (C) Semi-quantification of tumor volume in U251, 5310 GSCs alone and with hUCBSC treatment was done as described in Materials and Methods. Data shown here are the mean + SD values of 6 animals from each group. (D) Immuno-cytochemical fluorescence analysis of U251, U87, 4910 and 5310 Non GSCs using CD133, CD44, Ki-67, Msi-1, Nestin, Sox2, Stro-1 and Tuj-1 markers. Inset pictures show DAPI (blue) staining. Bar = 100 µm. (n=3).
